# Patients' Attitudes Towards Deprescribing Differ Across Specific Cardiovascular and Diabetes Medication: A Survey Study Assessing Within‐Patient Differences

**DOI:** 10.1111/bcpt.70140

**Published:** 2025-11-14

**Authors:** Peter J. C. Stuijt, Stijn Crutzen, Mette Heringa, Jessica V. Hootsen, Barbara C. van Munster, Anita T. Wildeboer, Katja Taxis, Petra Denig

**Affiliations:** ^1^ Department of Clinical Pharmacy and Pharmacology University of Groningen, University Medical Center Groningen Groningen the Netherlands; ^2^ Rob Giel Research Center, University Center Psychiatry University Medical Center Groningen, University of Groningen Groningen the Netherlands; ^3^ Lentis Research Lentis Psychiatric Institute Groningen the Netherlands; ^4^ SIR Institute for Pharmacy Practice and Policy Leiden the Netherlands; ^5^ Department of Geriatrics Martini Hospital Groningen the Netherlands; ^6^ Department of Internal Medicine University of Groningen, University Medical Center Groningen Groningen the Netherlands; ^7^ Department of Primary and Long‐Term Care University of Groningen, University Medical Center Groningen Groningen the Netherlands; ^8^ Unit of Pharmaco‐Therapy, ‐Epidemiology, and –Economics, Faculty of Science and Engineering University of Groningen Groningen the Netherlands

**Keywords:** cardiovascular, deprescribing, diabetes, older adults, survey

## Abstract

**Background:**

Knowing whether patients' attitudes towards deprescribing differ by medication is important for implementing deprescribing in practice.

**Objective:**

To assess whether there are within‐patient differences in attitudes towards deprescribing the following cardiovascular and diabetes medications: statins, antihypertensives, sulfonylureas and insulins.

**Methods:**

We administered the revised Patient Attitudes Towards Deprescribing questionnaire to Dutch primary care patients. The ‘appropriateness’ and ‘concerns’ factors were adapted to measure medication‐specific attitudes. Pairwise comparisons of appropriateness and concerns factor scores were tested with Wilcoxon signed‐rank tests and corrected to control the false discovery rate.

**Results:**

Responses from 160 patients (median age: 79 years, 34% frail) were used for the comparisons. Appropriateness factor scores were higher for insulins compared to statins (*n* = 18, 3.9 versus 3.3, *p* < 0.031), antihypertensives (*n* = 21, 4.0 versus 3.6, *p* < 0.031) and sulfonylureas (*n* = 12, 3.8 versus 3.4, *p* < 0.031) and higher for sulfonylureas compared to antihypertensives (*n* = 26, 3.6 versus 3.4, *p* = 0.036) and statins (*n* = 27, 3.6 versus 3.2, *p* = 0.006). No statistical differences were found for the concerns factor scores.

**Conclusion:**

Given the observed differences in appropriateness attitudes, patients may be more positive towards deprescribing statins and antihypertensives as compared to sulfonylureas and particularly insulins. Healthcare providers should be aware that patients can experience medication‐specific barriers when discussing options for deprescribing.

## Plain Language Summary

This study explored whether older patients feel differently about stopping specific cardiovascular and diabetes medications. Using a questionnaire, 160 older adults shared their views on potentially deintensifying their statins, antihypertensives, sulfonylureas and insulins. Comparisons were made among small groups of patients using multiple of these medications. Patients considered their sulfonylureas and especially their insulins relatively more appropriate than their statins and antihypertensives, but did not have more concerns when stopping these medications. Clinicians should consider these differences when discussing options to deintensify treatment with patients to ensure a patient‐centred deprescribing process.

## 1 Introduction

Medication optimisation is especially relevant in older and frail adults using multiple medications to decrease medication burden and reduce the risk of adverse drug events, drug–drug interactions and related healthcare utilisation. Deprescribing, which includes medication discontinuation, dose decreases or switches to less potent and lower risk medication, is an important strategy for optimising medication use. This may include deprescribing of cardiovascular and diabetes medication, for which practice guidelines have been developed in the Netherlands as well as in a number of other countries [1, 2, 3]. A recent review showed that deprescribing can reduce the number of inappropriate medications, though the effects varied depending on the targeted medication [4]. Implementing deprescribing in practice is challenging, and the willingness of both healthcare providers and patients is essential for effective deprescribing [5]. Several studies focusing on deprescribing of cardiovascular and diabetes medication illustrated that patients can perceive this medication as essential and lack knowledge about potential benefits of deintensifying such medication [2, 6]. This limits their willingness to have this medication deprescribed [7]. On the other hand, experiencing side effects or having concerns about medication safety can increase such willingness [7, 8].

A questionnaire to assess patients' willingness and attitudes towards deprescribing was published in 2013, and revised and validated in 2016 [9, 10]. The number of studies using one of the versions of this questionnaire is rapidly increasing [11]. In most studies, the proportion of people agreeing with the general statement “If my doctor said it was possible, I would be willing to stop one or more of my medicines” is 80% or more [11]. Lower percentages were seen among populations without polypharmacy or including people under 65 years of age [11]. Some studies indicate that other patient characteristics, such as general concerns about medication, comorbidity or frailty, may influence patients' attitudes towards deprescribing [12, 13, 14]. A limited number of studies used both medication‐specific and general statements and illustrated that willingness depends on whether or not specific medication is mentioned [15, 16, 17]. A previous study, focussing on cardiovascular and diabetes medication, showed that older patients on polypharmacy had more positive attitudes towards stopping statins as compared to stopping antihypertensives, and that they appeared to be more reluctant to stop insulin as compared to stopping other diabetes or cardiovascular medication [18]. Moreover, patients' willingness to discontinue specific medication may differ from their willingness to reduce the dose of such medication [19]. For example, more patients reported willingness to try stopping statins as compared to reducing the dose of statins, whereas for insulin this was the opposite [18].

When patients' attitudes towards deprescribing depend on specific medication, asking about attitudes in general is insufficient to support patient‐centred deprescribing. So far, little research has been done in patients taking multiple drugs to explore within‐patient differences in attitudes towards deprescribing different specific medications they use. The aim of this study is to assess whether there are differences in older patients' attitudes towards deprescribing cardiovascular and diabetes medication for which recommendations to deprescribe have been made: statins, antihypertensives, sulfonylureas and insulins, to build on the previously conducted study [18]. Our main objective is to assess whether there are within‐patient differences in these attitudes, which can thus be attributed to the attitudes towards specific medication classes. Additionally, we want to assess whether similar differences in medication‐specific attitudes can be observed between patients using different medication classes, who may differ in other characteristics.

## 2 Methods

### 2.1 Study Design, Setting and Participants

A cross‐sectional study was conducted to assess within‐patient differences in deprescribing attitudes comparing the following specific medication classes: (1) statins versus antihypertensives, (2) statins versus sulfonylureas, (3) statins versus insulins, (4) antihypertensives versus sulfonylureas, (5) antihypertensives versus insulins and (6) sulfonylureas versus insulins. This was done among subpopulations of patients using both medication classes for each comparison. The study was conducted in accordance with the Basic & Clinical Pharmacology & Toxicology policy for experimental and clinical studies [20]. The between‐patient differences were assessed by comparing attitudes across all patients using the relevant medication classes, as has been done before [18]. It should be noted that it may include both between‐patient and within‐patient data, since patients can use more than one medication class.

For this study, we used data collected with two baseline questionnaires between the first of December 2023 and the twelfth of February 2025 for the cluster‐randomized CO‐DEPRESCRIBE trial (ClinicalTrials.gov registered, identifier: NCT05507177). The objective of this trial is to evaluate the effects of a communication training program for healthcare provider teams about deprescribing of cardiovascular or diabetes medication in Dutch primary care [21]. All patients in the trial received a clinical medication review. It is common in the Netherlands that community pharmacists together with general practitioners conduct such reviews for older people on polypharmacy. For the trial, local teams of staff from community pharmacies and/or general practices recruited people of 75 years and older exposed to polypharmacy, who were eligible for a clinical medication review [21]. In addition, they should be prescribed at least one of the following treatments, which could make them eligible for deprescribing: a statin, and/or two or more antihypertensives, and/or a sulfonylurea, and/or one or more insulins and/or any two non‐insulin glucose‐lowering medications. Patients not able to answer questionnaires in Dutch were excluded. Informed consent was obtained by the researchers.

### 2.2 Outcomes and Background Variables

An adapted version of the validated Dutch version of the revised Patients' Attitudes Towards Deprescribing (rPATD) questionnaire was administered [10, 22]. The rPATD questionnaire consists of two global items and four factors each containing five items, all scored on a 5‐point Likert scale ranging from 1 = *strongly disagree* to 5 = *strongly agree*. Factor scores were calculated in case of no missing item scores by dividing the factor score (ranging from 5–25) by 5 (the number of items). The ‘appropriateness’ and ‘concerns’ factors were made medication specific, as was previously done [18, 23]. For this, the phrases “one or more of my medicines” were replaced by “my statin”, or “one or more of my antihypertensives”, or “my sulfonylurea”, or “my insulin”. As the questionnaire was intended to be administered during baseline and follow‐up in the CO‐DEPRESCRIBE trial, two appropriateness items and two concerns items were slightly adapted, so that identical items could be used for baseline and follow‐up administration (Appendix S1). To illustrate, the appropriateness item “I would like to try stopping …” was rephrased to “I am positive about stopping …”. The ‘burden’ and ‘involvement’ factors were kept unchanged, focusing on general attitudes. In the Netherlands, clinical medication reviews are typically initiated by the community pharmacist. To reflect this practice, the following global item was added: “If my community pharmacist said it would be possible, I would be willing to stop one or more of my regular medicines” (Appendix S1). To keep the number of items similar, the global question on satisfaction with current medication was deleted.

For the assessment of within‐patient differences in medication‐specific attitudes, the primary outcomes are the scores for the medication‐specific appropriateness and concerns factors. These are also the outcomes for assessing the between‐patient differences in medication‐specific attitudes. Additionally, scores on the individual medication‐specific appropriateness and concerns items are reported to provide insight into underlying differences. Scores for the two global items (“I would be willing to stop …”) and the items of the burden and involvement factors are presented to describe general attitudes in the study population towards deprescribing. Other descriptive variables are the following: age and sex (female/male) as extracted from the primary care information system, and self‐reported living situation (living alone or not) and frailty scores (assessed with the Tilburg Frailty Indicator, see Data Collection) [24].

### 2.3 Data Collection

Two baseline questionnaires were administered within an intended time span of 14 days prior to the first consultation for the clinical medication review. The first contained the general rPATD items (global items, involvement and burden items), and was administered via paper‐based mail or e‐mail with a link to the online questionnaire, unless people preferred administration via telephone (e.g., in case of impaired vision). To enable clarifications regarding the types of medication people were using, the second questionnaire with medication‐specific items was administered via telephone interviews, unless people preferred online or paper‐based administration (e.g., because of hearing problems). The second questionnaire contained the Tilburg Frailty indicator, a question about living situation and the medication‐specific rPATD items for the appropriateness and concerns factors. For the administration of the medication‐specific rPATD items, the patients were first asked which cardiovascular and/or diabetes medication they used. The interviews were conducted by one of five interviewers who were trained by PS and/or JH. Based on a feasibility pilot, an instruction—including a list of all medications of interest—was made to support the interviewers in assessing relevant medication used, and all interviewers were familiarized and trained with this instruction. In case of online or paper‐based administration, examples of generic names of the commonly used medicines were provided (Appendix S2). Next, the medication‐specific rPATD items were presented for each of the medication classes used by a patient (i.e., using a statin, two or more antihypertensives, a sulfonylurea and/or one or more insulins). When a person did not respond to the telephone or paper‐based administration, a new appointment for completing the questionnaire was made. In case of online administration, up to two reminders were sent.

### 2.4 Analyses

Descriptive statistics were presented separately for the total study population, that is, including all patients who answered at least one medication‐specific item for any of the four medication classes of interest, and for the subpopulation of patients who used at least two out of these four medication classes, to be included in the within‐patient analyses. Response categories ‘strongly (dis)agree’ were merged with the respective ‘(dis)agree’ categories for presenting the response distributions of the general items. The medication‐specific appropriateness item scores were reversed, with higher scores indicating that patients considered the medication more appropriate. Similarly, higher concern item scores indicate stronger concerns about deprescribing the medication. Study data were managed using REDCap electronic data capture tools hosted at the University Medical Center Groningen and analysed using R version 4.4.3 [25, 26].

#### 2.4.1 Within‐Patient Analyses

For the main objective, within patient comparisons of attitudes between different medication classes were made at the factor level for appropriateness and concerns. Pairwise deletion was performed to deal with missing data for testing. Differences in factor scores were tested with Wilcoxon signed‐rank tests. The rank‐biserial correlation (*r*) was calculated and used as effect size, reflecting the standardized difference between positive versus negative rank sums while neglecting tied ranks [27]. For example, an *r* of 0.50 reflects that 75% of the rank sum of all non‐zero differences for a comparison was in favour of Drug A, while the remaining 25% of the rank sum of non‐zero differences was in favour of Drug B. To control for chance findings due to testing six pairwise comparisons regarding the appropriateness and the concerns factor scores, the Benjamini–Hochberg correction was applied [28]. With six comparisons, the alpha was set to 0.0083 (i.e., 0.05/6) to indicate statistical significance for the first comparison with increasing values for alpha for each subsequent comparison. To give insight into differences in underlying items of the appropriateness and concerns factors for all six comparisons, descriptive comparisons are presented with effect sizes.

#### 2.4.2 Analyses of Between‐Patient Differences

For the assessment of between‐patient differences in medication‐specific attitudes, descriptive comparisons were made in the total study population of patients using one or more of the medication classes. Since a patient can use medication from more than one medication class and thus belong to multiple between‐patient populations, no independent statistical testing for these comparisons was conducted.

### 2.5 Ethics Statement

The Medical Ethics Review Committee of the University Center of Groningen has assessed that the CO‐DEPRESCRIBE study does not fall within the scope of the Dutch Medical Research Involving Human Participants Act (WMO). The study received approval as a non‐WMO study from the Central Ethics Review Board of the UMCG (number 202200038). Patients were included after they had received information about the study and had given their informed consent to participate.

## 3 Results

In total, 310 patients were included in the CO‐DEPRESCRIBE study. The questionnaire containing the medication‐specific rPATD items was not administered to 17 patients, as the medication review was never scheduled in 12 patients, and the timely administration by telephone failed in five patients. From the remaining 293 patients, 10 patients indicated not using any of the medications of interest. For two patients preferring paper‐based questionnaire administration and one preferring administration via e‐mail, no responses were received. Therefore, the total study population included 280 patients, some of whom only used one of the medications of interest. Their median age was 79 years, 53% were female, and 39% were classified as being frail. Most were included for using a statin and/or two or more antihypertensives (Table 1). Overall, 87% of the patients (strongly) agreed to the statement “if my doctor said it was possible I would be willing to stop one or more of my regular medicines” (Table 2). This general willingness was 45% for the statement of the community pharmacist saying it was possible, with 20% having a neutral response. For all five items on involvement, the majority of patients (strongly) agreed, indicating a desire to be involved. Regarding general burden items, the majority indicated that they felt they were taking a large number of medicines but that taking and using their medication was not inconvenient or burdensome (Table 2).

**TABLE 1:** Background characteristics for total study population and among those using at least two of the medication classes of interest.

Characteristic	Total study population (*n* = 280)		Using at least two medication classes (*n* = 160)	
Age in years, median (IQR)	79 (77–83)	(35 missing)	79 (77–82) (22 missing)	
Female	53%		53%	
Living alone	33%		35%	
TFI score ≥ 5	39%	(3 missing)	34%	
Medication use				
Using statins	69%	(*n* = 194)	91%	(*n* = 145)
Using ≥ 2 antihypertensives	75%	(*n* = 210)	91%	(*n* = 146)
Using sulfonylureas	15%	(*n* = 43)	24%	(*n* = 38)
Using insulins	12%	(*n* = 33)	19%	(*n* = 31)

Abbreviations: IQR = Inter Quartile Range; *n* = number of patients; TFI = Tilburg Frailty Indicator.

**TABLE 2:** General attitudes towards deprescribing for the total study population.

Revised attitudes towards deprescribing items	*n*	(Strongly) disagree (%)	Neutral (%)	(Strongly) agree (%)
If my doctor said it was possible I would be willing to stop one or more of my regular medicines	279	3	10	87
If my community pharmacist said it was possible I would be willing to stop one or more of my regular medicines	275	35	20	45
*Involvement items*				
I like to be involved in making decisions about my medicines with my doctor(s)	278	5	3	92
I have a good understanding of the reasons I was prescribed each of my medicines	279	7	11	82
I like to know as much as possible about my medicines	277	5	22	73
I always ask my doctor, pharmacist or other healthcare professional if there is something I do not understand about my medicines	277	6	17	77
I know exactly what medicines I am currently taking, and/or I keep an up‐to‐date list of my medicines	278	9	8	83
*Burden items*				
I feel that I am taking a large number of medicines	276	10	37	53
Taking my medicines every day is very inconvenient	276	68	24	8
I spend a lot of money on my medicines	270	47	33	20
Sometimes I think I take too many medicines	269	29	39	32
I feel that my medicines are a burden to me	275	70	23	7

*Note: n* = number of responses.

For the within‐patient comparisons, 160 patients were included who used two or more of the medication classes of interest. Their median age was also 79 years and 53% were female (Table 1). These characteristics were quite similar in the subpopulations included in each within‐patient comparison, except for comparisons involving insulin which included more patients with a higher age and being female (Appendix S3). The percentages of frail patients varied and seemed somewhat higher for comparisons of multiple antihypertensives with diabetes medication. The general willingness to stop medication among these 160 patients was similar as observed in the total population, with 90% agreeing to be willing to stop if the doctor said it was possible and 45% agreeing when the community pharmacist would say this (Appendix S4).

### 3.1 Within‐Patient Differences in Attitudes About Appropriateness and Concerns

For five of the six pairwise comparisons between medication classes, statistically significant differences in appropriateness scores were seen (Table 3). For the comparison between statins and multiple antihypertensives, there was no significant difference. The appropriateness score was higher for insulins compared to all other medication classes, reflecting a relatively greater belief in the appropriateness of insulins. Although the differences in median factor scores were relatively small, ranging from 0.4 to 0.6, the absolute values of the effect sizes were high for these comparisons, with few ties at the individual patient level. The appropriateness score was also higher for sulfonylureas when compared to statins and multiple antihypertensives, with differences in median factor scores ranging from 0.2 to 0.4. Looking at the underlying items, patients agreed particularly more strongly to the items “I am positive about stopping my … to see how I feel without it” for their statin as compared to their insulin or sulfonylurea, with differences of 2 on the 5‐point scale in median scores (Appendix S5). Notably, for the item “I believe my … may be currently giving me side effects” no differences were seen for any of the comparisons.

**TABLE 3:** Within‐patient differences in scores for appropriateness and concerns regarding different medication classes.

		*n* ^a^	Median (IQR) of 1st medication	Median (IQR) of 2nd medication	*p*	Non‐tied n^b^	*r*
Factor	Comparison						
Appropri‐ateness	Statins versus antihypertensives	126	3.2 (2.8–3.8)	3.2 (2.8–3.8)	0.3778	96	−0.15
	Statins versus sulfonylureas	27	3.2 (2.6–3.5)	3.6 (3.2–4.0)	0.0058^c^	23	−0.48
	Statins versus insulin	18	3.3 (2.8–3.6)	3.9 (3.6–4.2)	0.0015^c^	14	−0.86
	Antihypertensives versus sulfonylureas	26	3.4 (2.8–3.8)	3.6 (3.2–3.8)	0.0357^c^	21	−0.24
	Antihypertensives versus insulins	21	3.6 (3.2–4.0)	4.0 (3.6–4.0)	0.0306^c^	17	−0.53
	Sulfonylureas versus insulins	12	3.4 (2.8–3.8)	3.8 (3.6–4.0)	0.0140^c^	11	−0.81
Concerns	Statins versus Antihypertensives	109	2.6 (2.2–3.0)	2.8 (2.4–3.0)	0.4316	73	−0.15
	Statins versus sulfonylureas	24	2.5 (2.2–2.9)	2.8 (2.6–3.0)	0.0248	17	−0.53
	Statins versus insulin	16	2.7 (2.4–2.8)	2.8 (2.8–3.2)	0.0170	9	−0.78
	Antihypertensives versus sulfonylureas	24	2.6 (2.6–3.0)	2.8 (2.4–3.1)	0.9183	19	−0.16
	Antihypertensives versus insulins	20	2.8 (2.6–3.0)	2.9 (2.8–3.2)	0.0170	15	−0.73
	Sulfonylureas versus insulins	12	2.7 (2.0–3.1)	2.8 (2.6–2.9)	0.3398	9	−0.11

*Note: n* = number; IQR = Inter Quartile Range.

^a^ *n* = number of paired observations with valid data on all individual items. Lower numbers for concerns factor due to more missings particularly on the item “I have had a bad experience when stopping …” (Appendix S6).

^b^ Non‐tied *n* = number of paired observations for which the paired scores were non‐identical.

^c^ Marks statistical significance after Benjamini–Hochberg correction, applied separately for appropriateness and concerns factor score comparisons. *p*‐Values were ranked from low to high and respectively compared to adjusted alpha levels of 0.0083, 0.0167, 0.0250, 0.0333, 0.0417 and 0.0500.

No statistically significant differences were found for any of the concerns factor scores after applying Benjamini–Hochman correction for multiple testing (Table 3). Regarding the underlying items, the majority of patients agreed with the concerns items “I would be reluctant to stop my …” and “with stopping my … I would be worried about missing out on future benefits” (Appendix S5). The number of non‐identical within‐patient responses for these two items was higher than for the other three concerns items in all six comparisons. Moreover, based on the medians and the distribution of responses, more patients responded somewhat more positively to these two items for insulins as compared to statins and (“one or more”) antihypertensives, with high effect sizes (Appendix S5). This also applied to the comparison between sulfonylureas and statins. On the other hand, most patients disagreed with the other three concern items, regardless of the specific medication class. That is, few had bad experiences with stopping, or getting stressed whenever changes are made, or feeling that the healthcare provider is giving up on them when making a recommendation to stop the specific medication.

### 3.2 Differences in Attitudes Between Patients Using Different Medication Classes

**FIGURE 1:**
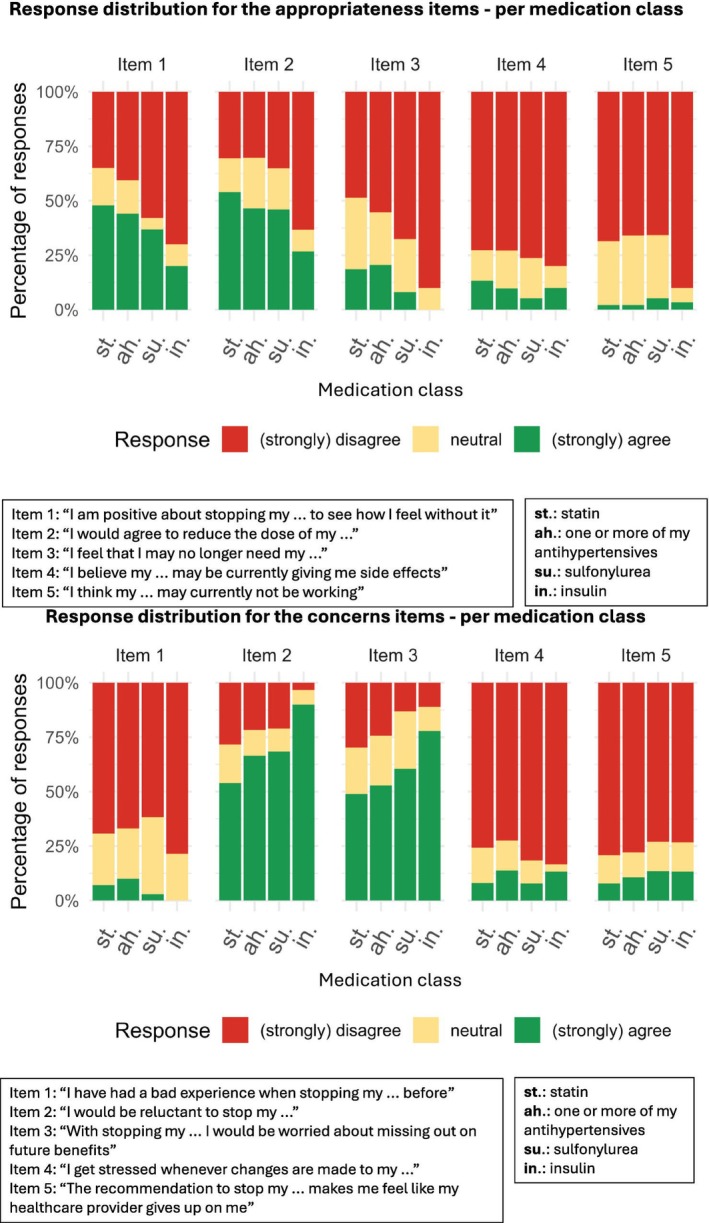
(A) Response distribution for appropriateness items among all patients using ≥ 2 medication classes (for included numbers, see Appendix S6). (B) Response distribution for concerns items among all patients using ≥ 2 medication classes (for included numbers, see Appendix S6).

The overall median scores for the appropriateness and concerns factors for the four medication classes are comparable to the respective scores used in the within‐patient comparisons across the different medication classes (Table 3 and Table 4). When comparing the responses to the underlying appropriateness items between the different medication classes, fewer patients using insulins agreed with the item “I am positive about stopping my …” (20%) as compared to sulfonylureas (37%), (“one or more”) antihypertensives (44%) or statins (48%) (Figure 1A, Appendix S6). Similarly, fewer patients using insulins agreed with the items “I would agree to reduce the dose of my …”, “I feel that I may longer need my …” or “I think that my … may currently not be working”.

**TABLE 4:** Overall appropriateness and concerns factor scores per medication class.

Factor	Medication class	*n* ^a^	Median score	IQR
Appropriateness	Statin	179	3.2	2.8–3.8
	Antihypertensives	203	3.2	2.8–3.8
	Sulfonylurea	42	3.6	3.2–4.0
	Insulin	32	4.0	3.6–4.1
Concerns	Statin	156	2.6	2.2–3.0
	Antihypertensives	181	2.8	2.4–3.0
	Sulfonylurea	39	2.8	2.4–3.1
	Insulin	27	2.8	2.8–3.1

*Note: n* = number; IQR = Inter Quartile Range.

^a^ *n* = number of observations with valid data on all individual items. Lower numbers for concerns factor due to more missings particularly on the item “I have had a bad experience when stopping …” (Appendix S6).

Regarding the concerns items, reluctance to stop with insulins was high (90%) compared to sulfonylureas (68%), (one or more) antihypertensives (66%) and statins (54%) (Figure 1B, Appendix S6). Similarly, being worried about missing out on future benefits was high for insulins (78%) compared to sulfonylureas (61%), (‘one or more’) antihypertensives (53%) and statins (49%).

## 4 Discussion

The appropriateness factor of the patients' attitudes towards deprescribing showed small but statistically significant differences across the specific medication classes for which the attitudes were assessed. Patients perceived the appropriateness of their insulins higher than other medication classes. Likewise, patients perceived the appropriateness of their sulfonylureas slightly higher than their antihypertensives or statins. The magnitude of the median differences ranged from 0.2 to 0.6 but in some of the underlying items larger differences were observed. We found no statistically significant differences in the concerns factor scores between the different medication classes, after correcting to control for chance findings. However, differences could be seen in two of the underlying concerns items, where particularly more patients had concerns regarding stopping their insulins as compared to the other classes and sulfonylureas as compared to statins, respectively. These results from within‐patient comparisons across different medication classes appeared to align with those from between‐patient comparisons.

The finding that patients rated the appropriateness of their insulin higher than that of the other medication classes is in line with the between‐patient comparisons in the previously conducted survey study [18]. Often, healthcare providers need to convince patients with Type 2 diabetes to initiate insulin, supporting the belief that insulin cannot be stopped [29]. The regular monitoring of glucose levels after insulin initiation might strengthen the belief that a patient needs continuous use of the insulin [30]. The observed differences in perceived appropriateness between the medication classes could also be related to both differences in a patient's ability to perceive (short‐term) effectiveness of the drug used and the perceived severity of disease. An interview study found that patients with Type 2 diabetes often considered their diabetes medication more important than other cardiovascular medication, with lipid‐lowering medication considered the least important in cardiovascular risk management [31]. Meanwhile, dyslipidaemia was frequently viewed as a condition that could be managed effectively by lifestyle modifications alone [31]. In the previous study, patients using both a statin and one or more antihypertensives considered their antihypertensive(s) to be more appropriate than their statin [18]. Among patients using both a statin and two or more antihypertensives in our study; however, no clear differences were seen in their attitudes towards deprescribing these medications. This discrepancy in findings might be due to differences in patient populations. Our patient population was at least 75 years of age and selected for being prescribed specific cardiovascular or diabetes medication, which could make them eligible for deprescribing such medication. We focused on attitudes towards deprescribing insulins and sulfonylureas, which are considered the most relevant glucose‐lowering medication classes for deprescribing, given their high risks of causing hypoglycaemic events [32]. Similarly, we focused on attitudes towards deprescribing antihypertensive medication among patients who used two or more of these medications, making them more at risk of adverse outcomes [33]. The higher age and use of more antihypertensives, as compared to the previous study, might explain why the appropriateness scores for antihypertensives were somewhat lower than observed in the previous study (median 3.2 vs. 3.7) [18]. In this study, the results of the within‐patient comparisons of medication‐specific attitudes appeared rather similar to those for the between‐patient comparisons. This could mean that the differences in medication‐specific attitudes are either not influenced by differences in patient characteristics or that the samples of patients using these medication classes are rather similar regarding characteristics that may influence patients' attitudes towards deprescribing.

The high general willingness to stop one or more regular medications if the doctor said it would be possible seen in this study is consistent with previous findings [11, 18]. In case the pharmacist would say it was possible, only half the number of patients would agree. Notably, these questions do not capture the nuance of proposals for deprescribing being made in medication reviews that are conducted jointly by the general practitioner and the pharmacist. Previously, the feasibility and effectiveness of pharmacist‐led collaborative approaches have been established and integration of pharmacists in local primary care teams is recommended to support deprescribing [34, 35]. Nonetheless, community pharmacists have reported lower perceived patient trust compared to general practitioners and nurse practitioners regarding deprescribing cardiovascular or diabetes medication [36].

### 4.1 Strengths and Limitations

To our knowledge, this is the first study performing within‐patient comparisons of medication‐specific attitudes towards deprescribing for different medication classes. To ascertain which medication a patient used and be able to prevent potential misunderstandings for the administration of the medication‐specific appropriateness and concerns items, we administered these via telephone. We were able to administer medication‐specific rPATD items to 280 of the 310 patients in the CO‐DEPRESCRIBE trial. Additionally, we adjusted for multiple comparisons in our main analysis using the Benjamini–Hochberg procedure to preserve statistical power while controlling the proportion of false positive results [28]. Some limitations should be considered when interpreting the results. Firstly, the adaptations that we made to administer medication‐specific items have not been validated but appear not to have led to unexpected responses. Secondly, the included study population participated in a cluster‐randomized trial in which some will receive a clinical medication review about possibilities for deprescribing. Therefore, the results might not be representative for all older adults using cardiometabolic medication, who might be less willing to have such medication deprescribed. Thirdly, for some comparisons the numbers of patients were small with some shifts in age, sex and frailty. There might be other differences in patient characteristics for which we did not have data, like socioeconomic or functional status or total number of medications used. Nonetheless, previous studies showed inconsistent associations between attitudes towards deprescribing and characteristics like age, sex, number of medications, educational level or frailty [11, 12, 15, 37]. We observed statistically significant differences in appropriateness factor scores between different medication groups, but whether these differences are associated with differences in actual rates of deprescribing remains unknown. Finally, the between‐patient comparisons are confounded by the inclusion of within‐patient data, limiting firm conclusions regarding the influence of other patient characteristics on medication‐specific attitudes.

### 4.2 Implications for Research and Clinical Practice

Our study adds to previous studies that the observed differences in attitudes towards deprescribing specific cardiometabolic medication classes can exist within one patient [15, 18]. Although we do not know yet how meaningful the observed differences in factor scores are, the differences by medication class observed for some of the appropriateness items could be relevant for clinical practice. Identifying patients' attitudes towards deprescribing in clinical practice thus requires medication‐specific questions, which are needed to facilitate a patient‐centred approach in weighing options and prioritizing potential interventions. Previous results indicate that older patients who may be more eligible for deprescribing due to frailty or using multiple glucose‐lowering drugs may be less willing to do so [12, 13, 15, 23]. This highlights the importance of having timely discussions with patients and/or caregivers about the pros and cons of continuing and discontinuing medication, which has been stressed before [2].

The finding that the majority of patients agreed with two of the concern items but disagreed with the other three concern items regardless of the medication class, might be an indication that the adaptations to make the factor medication‐specific have compromised the validity of the factor. The items “I get stressed whenever changes are made to my …” and “I have had a bad experience when stopping my … before” might predominantly capture sporadic occurrences of medication changes and stops rather than reflecting medication‐specific attitudes. The item “the recommendation to stop my … makes me feel like my healthcare provider gives up on me” might be more reflective of patients' generalized trust in their local healthcare providers rather than a medication‐specific notion. To evaluate any adaptations, factor analyses could be performed to assess factor construct validity, as has previously been done in a number of translation validation studies [38, 39, 40].

Several studies have reported on patient and/or caregiver barriers and facilitators for deprescribing cardiovascular and/or diabetes medication, providing both potential reasons for unwillingness and enabling perspectives to deprescribe cardiometabolic medication [6, 7, 30, 41]. Of note, in line with these studies, general attitudes towards deprescribing can be multifaceted, with people on the one hand feeling that they are taking a lot of medication but not feeling that their medicines are a burden to them. Lack of perceived medication burden can be a barrier to deprescribing. To advance deprescribing in practice, more work should be done on the relationship between the different rPATD (factor) scores, the extent of patients' engagement in deprescribing, and actual rates of deprescribing. Studies combining changes or differences in rPATD scores with process‐related or actual deprescribing outcomes can help to identify which attitudes are most relevant to address before and during patient consultations about deprescribing. Finally, little is currently known about the extent to which patients' attitudes towards deprescribing might change due to interventions. In one study, no significant changes were observed following a medication review intervention [42]. Two trials (DeprescriPP and CO‐DEPRESCRIBE) measuring attitudes before and after an intervention focusing on deprescribing are currently ongoing [21, 43].

## 5 Conclusion

Patients in our study were somewhat more positive about the appropriateness of their sulfonylureas and particularly their insulins as compared to their statins and antihypertensives. Given these differences, patients may be more positive towards deprescribing statins and antihypertensives as compared to sulfonylureas and particularly insulins. During the process of deprescribing, healthcare providers should assess potential medication‐specific attitudes that need to be addressed, to allow for a patient‐centred deprescribing approach.

## Conflicts of Interest

The authors declare no conflicts of interest.

## Supporting information


**Appendix S1:** Adapted rPATD for attempting to measure medication‐specific attitudes towards deprescribing.

**Appendix S2:** Introduction of medication‐specific items in case of online or paper‐based administration.

**Appendix S3:** Age and sex distribution for all subgroup comparisons (pairwise deletion when response on item was missing).

**Appendix S4:** Generic attitudes towards deprescribing among patients using at least two of the selected medication classes, only for patients whose data were used for the within‐patient analysis.

**Appendix S5:** Within‐patient differences in medication‐specific items from appropriateness and concerns factors.

**Appendix S6:** (A) Appropriateness‐ and concerns‐responses about deprescribing statin (with medication‐specific revised Patients' Attitudes Towards Deprescribing questions).
**Appendix S6:** (B) Appropriateness‐ and concerns‐responses about deprescribing one or more antihypertensives (with medication‐specific revised Patients' Attitudes Towards Deprescribing questions).
**Appendix S6:** (C) Appropriateness‐ and concerns‐responses about deprescribing sulfonylurea (with medication‐specific revised Patients' Attitudes Towards Deprescribing questions).
**Appendix S6:** (D) Appropriateness‐ and concerns‐responses about deprescribing insulin (with medication‐specific revised Patients' Attitudes Towards Deprescribing questions).

## Data Availability

The data that support the findings of this study are available on request from the corresponding author. The data are not publicly available due to privacy or ethical restrictions.
