# Supplementary material for: Patients' Attitudes Towards Deprescribing Differ Across Specific Cardiovascular and Diabetes Medication: A Survey Study Assessing Within‐Patient Differences
Source: Basic Clin Pharmacol Toxicol. 2025 Nov 14;137(6):e70140. doi: 10.1111/bcpt.70140 (PMC12617390; doi:10.1111/bcpt.70140)
Supplement: Supplementary file 1 — Appendix S1: Adapted rPATD for attempting to measure medication‐specific attitudes towards deprescribing. [file BCPT-137-0-s002.docx]

Appendix 1. Adapted rPATD for attempting to measure medication-specific attitudes towards deprescribing

| **Questions with changes, blue = adapted text, ~~red~~ = removed text, XXX=specific medication name** |
| --- |
| ***Global items*** |
| If my doctor said it was possible, I would be willing to stop one or more of my medicines |
| If my pharmacist ~~doctor~~ said it was possible, I would be willing to stop one or more of my medicines* |
| ***Appropriateness items*** |
| I ~~would like to try~~ am positive about stopping ~~one of~~ my XXX ~~medicines~~ to see how I feel without it. |
| I ~~would like my doctor~~ would agree to reduce the dose of ~~one or more of~~ my XXX ~~medicines~~. |
| I feel that I ~~may be taking one or more medicines that~~ no longer need my XXX. |
| I believe ~~one or more of~~ my XXX ~~medicines~~ may be currently giving me side effects. |
| I think ~~one or more of~~ my ~~medicines~~ XXX may not be working. |
| ***Concerns items*** |
| I have had a bad experience when stopping ~~a medicine~~ my XXX before. |
| I would be reluctant to stop ~~a medicine that I had been taking for a long time~~ my XXX. |
| ~~If one of~~ With stopping my XXX  ~~medicines was stopped~~ I would be worried about missing out on future benefits |
| I get stressed whenever changes are made to my XXX ~~medicines~~ |
| ~~If my doctor recommended~~ The recommendation to stop~~ping~~ my XXX ~~a medicine I would~~ makes me feel ~~that he/she was~~ like my healtcare proviver gives~~ing~~ up on me |

* As the clinical medication reviews in the CO-DEPRESCRIBE trial are typically initiated by the community pharmacist, we wanted to be able to measure willingness to deprescribe if the community pharmacist would indicate this possibility. We choose to replace the global item “Overall, I am satisfied with my current medicines” with the pharmacist item, as we deemed the global item about overall medication satisfaction less relevant with the administration of medication-specific items and wanted to keep the number of items similar.
