# Supplementary material for: Patients' Attitudes Towards Deprescribing Differ Across Specific Cardiovascular and Diabetes Medication: A Survey Study Assessing Within‐Patient Differences
Source: Basic Clin Pharmacol Toxicol. 2025 Nov 14;137(6):e70140. doi: 10.1111/bcpt.70140 (PMC12617390; doi:10.1111/bcpt.70140)
Supplement: Supplementary file 2 — Appendix S2: Introduction of medication‐specific items in case of online or paper‐based administration. [file BCPT-137-0-s004.docx]

# Appendix 2. Introduction of medication-specific items in case of online or paper-based administration.

**The following questions are about how you think about your medications.**

**First, we would like to ask whether you use medications for diabetes or blood sugar, or for your blood pressure or cholesterol.** **Please indicate which types of medications listed below you are using.**

**Please check all the answers that apply to you.**

***If you know the name (brand) of the medication, please write it down as well.
For each type of medication, a few examples are provided.***

Insulin, namely…………………………………………………………………………….……………

*For example: insuline aspart, insuline degludec of insuline glargine*

Sulfonylurea (SU) derivative (for diabetes, to lower your blood sugar), namely……………………………………………………………………………………………………………

*For example:* gliclazide*, glibenclamide, tolbutamide of glimepiride*

**2 or more** blood pressure-lowering medications, namely………………………..……………………………

……………………………………………………………………………………………………………………………………………………………………………………………………………………………………………………

*A combination of two or more of the following medications, for example: hydrochlorothiazide, amlodipine, lisinopril, losartan, or metoprolol*

Statin (cholesterol-lowering medication), namely
………………………………………………………………………………………………………………………..

*For example: simvastatin, pravastatin, fluvastatin, atorvastatin, rosuvastatin, or pitavastatin*

Would you like to elaborate on your answer? You can do so below. …………………………………………………………………………………………….…………………

……………………………………………………………………………………………………………….

**For each group of medications that you use, we would like to ask you a few questions. You only need to answer the questions for the medications you are using.**
