# Supplementary material for: Patients' Attitudes Towards Deprescribing Differ Across Specific Cardiovascular and Diabetes Medication: A Survey Study Assessing Within‐Patient Differences
Source: Basic Clin Pharmacol Toxicol. 2025 Nov 14;137(6):e70140. doi: 10.1111/bcpt.70140 (PMC12617390; doi:10.1111/bcpt.70140)
Supplement: Supplementary file 3 — Appendix S3: Age and sex distribution for all subgroup comparisons (pairwise deletion when response on item was missing). [file BCPT-137-0-s006.docx]

# Appendix 3. Age and sex distribution for all subgroup comparisons (pairwise deletion when response on item was missing)

|  |  | n | Age (years), median (IQR) | Sex (% female) | Living alone (%) | Tilburg Frailty Indicator score ≥5, % |
| --- | --- | --- | --- | --- | --- | --- |
| Statin versus Antihypertensives | Appropriateness | 126 | 78.5 (76.0-81.2)  [18 missing] | 52% | 33% | 33% |
|  | Concerns | 109 | 79.0 (76.2-81.8)  [15 missing] | 53% | 32% | 32% |
| Statin versus Insulin | Appropriateness | 18 | 80.5 (77.2-82.5) [4 missing] | 67% | 28% | 28% |
|  | Concerns | 16 | 78.5 (77.0-81.0) [4 missing] | 69% | 31% | 31% |
| Statin versus Sulfonylurea | Appropriateness | 27 | 77.0 (76.0-81.0) [2 missing] | 56% | 37% | 37% |
|  | Concerns | 24 | 77.0 (76.0-81.0) [3 missing] | 54% | 33% | 38% |
| Antihypertensives versus Insulin | Appropriateness | 21 | 80.0 (78.0-82.0)  [4 missing] | 71% | 33% | 43% |
|  | Concerns | 20 | 80.0 (78.0-82.0)  [4 missing] | 75% | 35% | 45% |
| Antihypertensives versus Sulfonylurea | Appropriateness | 26 | 78.0 (76.0-82.5)  [3 missing] | 54% | 38% | 42% |
|  | Concerns | 24 | 78.0 (76.0-81.2)  [4 missing] | 54% | 33% | 42% |
| Insulin versus Sulfonylurea | Appropriateness | 12 | 80.0 (77.5-82.0)  [1 missing] | 83% | 17% | 33% |
|  | Concerns | 12 | 79.5 (77.2-81.0)  [2 missing] | 83% | 17% | 25% |

n = number of observations
