# Supplementary material for: Patients' Attitudes Towards Deprescribing Differ Across Specific Cardiovascular and Diabetes Medication: A Survey Study Assessing Within‐Patient Differences
Source: Basic Clin Pharmacol Toxicol. 2025 Nov 14;137(6):e70140. doi: 10.1111/bcpt.70140 (PMC12617390; doi:10.1111/bcpt.70140)
Supplement: Supplementary file 4 — Appendix S4: Generic attitudes towards deprescribing among patients using at least two of the selected medication classes, only for patients whose data were used for the within‐patient analysis. [file BCPT-137-0-s005.docx]

Appendix 4. Generic attitudes towards deprescribing among patients using at least two of the selected medication classes, only for patients whose data were used for the within-patient analysis.

| **revised Attitudes Towards Deprescribing item scores** | **n** | **(strongly) disagree (%)** | **Neutral (%)** | **(strongly) agree (%)** |
| --- | --- | --- | --- | --- |
| If my doctor said it was possible I would be willing to stop one or more of my regular medicines | 160 | 2 | 8 | 90 |
| If my community pharmacist said it was possible I would be willing to stop one or more of my regular medicines | 159 | 31 | 23 | 46 |
| *Involvement items* |  |  |  |  |
| I like to be involved in making decisions about my medicines with my doctor(s) | 160 | 3 | 5 | 92 |
| I have a good understanding of the reasons I was prescribed each of my medicines | 160 | 7 | 12 | 81 |
| I like to know as much as possible about my medicines | 159 | 5 | 26 | 69 |
| I always ask my doctor, pharmacist or other healthcare professional if there is something I don’t understand about my medicines | 160 | 7 | 19 | 74 |
| I know exactly what medicines I am currently taking, and/or I keep an up-to-date list of my medicines | 160 | 10 | 8 | 82 |
| *Burden items* |  |  |  |  |
| I feel that I am taking a large number of medicines | 159 | 10 | 34 | 56 |
| Taking my medicines every day is very inconvenient | 158 | 69 | 24 | 7 |
| I spend a lot of money on my medicines | 156 | 46 | 33 | 21 |
| Sometimes I think I take too many medicines | 155 | 30 | 35 | 35 |
| I feel that my medicines are a burden to me | 158 | 66 | 27 | 7 |

n = number of responses
