# Supplementary material for: Patients' Attitudes Towards Deprescribing Differ Across Specific Cardiovascular and Diabetes Medication: A Survey Study Assessing Within‐Patient Differences
Source: Basic Clin Pharmacol Toxicol. 2025 Nov 14;137(6):e70140. doi: 10.1111/bcpt.70140 (PMC12617390; doi:10.1111/bcpt.70140)
Supplement: Supplementary file 5 — Appendix S5: Within‐patient differences in medication‐specific items from appropriateness and concerns factors. [file BCPT-137-0-s003.docx]

# Appendix 5. Within-patient differences in medication-specific items from appropriateness and concerns factors

|  | n | Median (IQR) of 1^st^ medication | Median (IQR) of 2^nd^ medication | Non-tied n | *r* |  |
| --- | --- | --- | --- | --- | --- | --- |
| Statin versus Antihypertensives | | | | | | |
| I am positive about stopping my … to see how I feel without it | 129 | 3.0 (2.0-4.0) | 3.0 (2.0-4.0) | 52 | -.08 |  |
| I would agree to reduce the dose of my … | 129 | 2.0 (2.0-4.0) | 3.0 (2.0-4.0) | 55 | -.13 |  |
| I feel that I may no longer need my … | 127 | 3.0 (3.0-4.0) | 4.0 (3.0-4.0) | 67 | -.04 |  |
| I believe my … may be currently giving me side effects | 132 | 4.0 (3.0-4.0) | 4.0 (3.0-4.0) | 43 | -.07 |  |
| I think my … may currently not be working | 129 | 4.0 (3.0-4.0) | 4.0 (3.0-4.0) | 43 | .07 |  |
| I have had a bad experience when stopping my … before | 116 | 2.0 (2.0-3.0) | 2.0 (2.0-3.0) | 24 | -.08 |  |
| I would be reluctant to stop my … | 130 | 4.0 (2.0-4.0) | 4.0 (3.0-4.0) | 49 | -.22 |  |
| With stopping my … I would be worried about missing out on future benefits | 127 | 3.0 (2.0-4.0) | 3.0 (2.0-4.0) | 57 | -.09 |  |
| I get stressed whenever changes are made to my … | 125 | 2.0 (2.0-2.0) | 2.0 (2.0-3.0) | 22 | -.36 |  |
| The recommendation to stop my … makes me feel like my healthcare provider gives up on me | 128 | 2.0 (2.0-2.0) | 2.0 (2.0-2.0) | 25 | -.28 |  |
| Statin versus insulin | | | | | | |
| I am positive about stopping my … to see how I feel without it | 19 | 2.0 (2.0-4.0) | 4.0 (3.0-4.0) | 10 | -.80 |  |
| I would agree to reduce the dose of my … | 19 | 2.0 (2.0-4.0) | 4.0 (3.0-4.0) | 11 | -.45 |  |
| I feel that I may no longer need my … | 19 | 4.0 (2.5-4.0) | 4.0 (4.0-4.0) | 11 | -1.00 |  |
| I believe my … may be currently giving me side effects | 20 | 4.0 (4.0-4.0) | 4.0 (4.0-4.0) | 6 | .00 |  |
| I think my … may currently not be working | 19 | 4.0 (3.5-4.0) | 4.0 (4.0-5.0) | 10 | -1.00 |  |
| I have had a bad experience when stopping my … before | 17 | 2.0 (2.0-2.0) | 2.0 (2.0-2.5) | 3 | -.33 |  |
| I would be reluctant to stop my … | 20 | 4.0 (2.5-4.0) | 4.0 (4.0-4.0) | 12 | -1.00 |  |
| With stopping my … I would be worried about missing out on future benefits | 17 | 3.0 (2.5-4.0) | 4.0 (4.0-4.0) | 10 | -1.00 |  |
| I get stressed whenever changes are made to my … | 19 | 2.0 (2.0-2.0) | 2.0 (2.0-2.0) | 5 | .60 |  |
| The recommendation to stop my … makes me feel like my healthcare provider gives up on me | 19 | 2.0 (2.0-2.0) | 2.0 (2.0-2.0) | 5 | -.60 |  |
| Statin vs Sulfonylurea | | | | | | |
| I am positive about stopping my … to see how I feel without it | 28 | 2.0 (2.0-3.0) | 4.0 (2.0-4.0) | 18 | -.67 |  |
| I would agree to reduce the dose of my … | 27 | 2.0 (2.0-3.0) | 2.0 (2.0-4.0) | 12 | -.50 |  |
| I feel that I may no longer need my … | 27 | 3.0 (2.0-4.0) | 4.0 (3.5-4.0) | 19 | -.58 |  |
| I believe my … may be currently giving me side effects | 28 | 4.0 (3.0-4.0) | 4.0 (3.0-4.0) | 10 | -.40 |  |
| I think my … may currently not be working | 28 | 4.0 (3.0-4.0) | 4.0 (3.0-4.0) | 8 | .00 |  |
| I have had a bad experience when stopping my … before | 25 | 2.0 (2.0-3.0) | 2.0 (2.0-3.0) | 4 | -.50 |  |
| I would be reluctant to stop my … | 28 | 3.0 (2.0-4.0) | 4.0 (2.5-4.0) | 13 | -.69 |  |
| With stopping my … I would be worried about missing out on future benefits | 28 | 3.0 (2.0-4.0) | 4.0 (3.0-4.0) | 18 | -.67 |  |
| I get stressed whenever changes are made to my … | 27 | 2.0 (2.0-2.5) | 2.0 (2.0-2.0) | 5 | .60 |  |
| The recommendation to stop my … makes me feel like my healthcare provider gives up on me | 27 | 2.0 (2.0-2.0) | 2.0 (2.0-2.0) | 6 | -.67 |  |
| Antihypertensives versus Insulin | | | | | | |
| I am positive about stopping my … to see how I feel without it | 21 | 4.0 (2.8-4.0) | 4.0 (3.0-4.0) | 9 | -.33 |  |
| I would agree to reduce the dose of my … | 21 | 3.0 (2.0-4.0) | 4.0 (3.0-4.0) | 16 | -.50 |  |
| I feel that I may no longer need my … | 21 | 4.0 (3.0-4.0) | 4.0 (4.0-4.0) | 10 | -.80 |  |
| I believe my … may be currently giving me side effects | 21 | 4.0 (4.0-4.0) | 4.0 (4.0-4.0) | 4 | .50 |  |
| I think my … may currently not be working | 21 | 4.0 (3.8-4.0) | 4.0 (4.0-4.3) | 6 | -.67 |  |
| I have had a bad experience when stopping my … before | 20 | 2.0 (2.0-2.3) | 2.0 (2.0-2.3) | 4 | .00 |  |
| I would be reluctant to stop my … | 21 | 4.0 (3.0-4.0) | 4.0 (4.0-4.0) | 9 | -.78 |  |
| With stopping my … I would be worried about missing out on future benefits | 20 | 4.0 (2.8-4.0) | 4.0 (4.0-4.0) | 9 | -.78 |  |
| I get stressed whenever changes are made to my … | 20 | 2.0 (2.0-2.0) | 2.0 (2.0-2.0) | 5 | .20 |  |
| The recommendation to stop my … makes me feel like my healthcare provider gives up on me | 21 | 2.0 (2.0-2.0) | 2.0 (2.0-3.0) | 4 | -.50 |  |
| Antihypertensives versus Sulfonylurea | | | | | | |
| I am positive about stopping my … to see how I feel without it | 27 | 3.0 (2.0-4.0) | 4.0 (2.0-4.0) | 10 | -.60 |  |
| I would agree to reduce the dose of my … | 26 | 3.0 (2.0-4.0) | 3.0 (2.0-4.0) | 13 | -.08 |  |
| I feel that I may no longer need my … | 26 | 4.0 (2.5-4.0) | 4.0 (3.5-4.0) | 13 | -.54 |  |
| I believe my … may be currently giving me side effects | 27 | 4.0 (3.0-4.0) | 4.0 (4.0-4.0) | 9 | -.56 |  |
| I think my … may currently not be working | 27 | 4.0 (3.0-4.0) | 4.0 (3.0-4.0) | 8 | -.25 |  |
| I have had a bad experience when stopping my … before | 24 | 2.0 (2.0-3.0) | 2.0 (2.0-3.0) | 2 | .00 |  |
| I would be reluctant to stop my … | 27 | 4.0 (2.5-4.0) | 4.0 (3.0-4.0) | 12 | -.17 |  |
| With stopping my … I would be worried about missing out on future benefits | 27 | 3.0 (3.0-4.0) | 4.0 (3.0-4.0) | 14 | -.29 |  |
| I get stressed whenever changes are made to my … | 27 | 2.0 (2.0-2.0) | 2.0 (2.0-2.0) | 7 | .71 |  |
| The recommendation to stop my … makes me feel like my healthcare provider gives up on me | 27 | 2.0 (2.0-2.5) | 2.0 (2.0-2.0) | 4 | .00 |  |
| Insulin versus Sulfonylurea | | | | | | |
| I am positive about stopping my … to see how I feel without it | 13 | 4.0 (2.5-4.0) | 2.0 (2.0-4.0) | 6 | .67 |  |
| I would agree to reduce the dose of my … | 12 | 4.0 (3.0-4.0) | 2.0 (2.0-3.0) | 7 | .71 |  |
| I feel that I may no longer need my … | 12 | 4.0 (4.0-4.0) | 4.0 (3.0-4.0) | 7 | .71 |  |
| I believe my … may be currently giving me side effects | 13 | 4.0 (4.0-4.0) | 4.0 (3.5-4.0) | 4 | .50 |  |
| I think my … may currently not be working | 13 | 4.0 (4.0-4.0) | 4.0 (3.0-4.0) | 7 | .71 |  |
| I have had a bad experience when stopping my … before | 12 | 2.0 (2.0-2.0) | 2.0 (2.0-2.0) | 1 | -1.00 |  |
| I would be reluctant to stop my … | 13 | 4.0 (4.0-4.0) | 4.0 (2.0-4.0) | 6 | 1.00 |  |
| With stopping my … I would be worried about missing out on future benefits | 12 | 4.0 (4.0-4.0) | 4.0 (2.5-4.0) | 6 | .67 |  |
| I get stressed whenever changes are made to my … | 13 | 2.0 (2.0-2.0) | 2.0 (2.0-2.0) | 5 | -.60 |  |
| The recommendation to stop my … makes me feel like my healthcare provider gives up on me | 12 | 2.0 (2.0-2.0) | 2.0 (2.0-2.0) | 2 | .00 |  |

IQR, Inter Quartile Range; r, effect size. No corrections for an increased false discovery rate were made.
