# Supplementary material for: Patients' Attitudes Towards Deprescribing Differ Across Specific Cardiovascular and Diabetes Medication: A Survey Study Assessing Within‐Patient Differences
Source: Basic Clin Pharmacol Toxicol. 2025 Nov 14;137(6):e70140. doi: 10.1111/bcpt.70140 (PMC12617390; doi:10.1111/bcpt.70140)
Supplement: Supplementary file 6 — Appendix S6: (A) Appropriateness‐ and concerns‐responses about deprescribing statin (with medication‐specific revised Patients' Attitudes Towards Deprescribing questions). Appendix S6: (B) Appropriateness‐ and concerns‐responses about deprescribing one or more antihypertensives (with medication‐specific revised Patients' Attitudes Towards Deprescribing questions). Appendix S6: (C) Appropriateness‐ and concerns‐responses about deprescribing sulfonylurea (with medication‐specific revised Patients' Attitudes Towards Deprescribing questions). Appendix S6: (D) Appropriateness‐ and concerns‐responses about deprescribing insulin (with medication‐specific revised Patients' Attitudes Towards Deprescribing questions). [file BCPT-137-0-s001.docx]

Appendix 6A. Appropriateness- and concerns-responses about deprescribing **statin** (with medication-specific revised Patients’ Attitudes Towards Deprescribing questions)

| **Item** | **Sample** | **(strongly) disagree (%)** | **Neutral (%)** | **(strongly) agree (%)** |
| --- | --- | --- | --- | --- |
| **Appropriateness** |  |  |  |  |
| I am positive about stopping my statin to see how I feel without it | Total sample (n=185) | 35 | 16 | 49 |
|  | Using ≥ 2 medication classes (n=140) | 35 | 17 | 48 |
| I would agree to reduce the dose of my statin | Total sample (n=185) | 29 | 15 | 56 |
|  | Using ≥ 2 medication classes (n=141) | 30 | 16 | 54 |
| I feel that I may no longer need my statin | Total sample (n=184) | 48 | 30 | 22 |
|  | Using ≥ 2 medication classes (n=140) | 48 | 33 | 19 |
| I believe my statin may be currently giving me side effects | Total sample (n=188) | 70 | 15 | 15 |
|  | Using ≥ 2 medication classes (n=143) | 73 | 14 | 13 |
| I think my statin may currently not be working | Total sample (n=184) | 67 | 30 | 3 |
|  | Using ≥ 2 medication classes (n=140) | 69 | 29 | 2 |
| **Concerns about stopping** |  |  |  |  |
| I have had a bad experience when stopping my statin before | Total sample (n=164) | 68 | 26 | 6 |
|  | Using ≥ 2 medication classes (n=127) | 69 | 24 | 7 |
| I would be reluctant to stop my statin | Total sample (n=185) | 33 | 17 | 50 |
|  | Using ≥ 2 medication classes (n=141) | 28 | 18 | 54 |
| With stopping my statin I would be worried about missing out on future benefits | Total sample (n=184) | 31 | 22 | 47 |
|  | Using ≥ 2 medication classes (n=141) | 30 | 21 | 49 |
| I get stressed whenever changes are made to my statin | Total sample (n=179) | 75 | 17 | 8 |
|  | Using ≥ 2 medication classes (n=136) | 76 | 16 | 8 |
| The recommendation to stop my statin makes me feel like my healthcare provider gives up on me | Total sample (n=183) | 81 | 11 | 8 |
|  | Using ≥ 2 medication classes (n=139) | 79 | 13 | 8 |

n = number of responses

Appendix 6B. Appropriateness- and concerns-responses about deprescribing **one or more antihypertensives** (with medication-specific revised Patients’ Attitudes Towards Deprescribing questions)

| **Item** | | **Sample** | **(strongly) disagree (%)** | **Neutral (%)** | **(strongly) agree (%)** |
| --- | --- | --- | --- | --- | --- |
|  | **Appropriateness** | | | | |
| I am positive about stopping one or more of my antihypertensives to see how I feel without it | | Total sample (n=206) | 38 | 17 | 45 |
|  |  | Using ≥ 2 medication classes (n=143) | 41 | 15 | 44 |
| I would agree to reduce the dose of one or more of my antihypertensives | | Total sample (n=205) | 29 | 21 | 50 |
|  |  | Using ≥ 2 medication classes (n=142) | 31 | 23 | 46 |
| I feel that I may no longer need one or more of my antihypertensives | | Total sample (n=204) | 53 | 26 | 21 |
|  |  | Using ≥ 2 medication classes (n=141) | 55 | 24 | 21 |
| I believe one or more of my antihypertensives may be currently giving me side effects | | Total sample (n=207) | 72 | 16 | 12 |
|  |  | Using ≥ 2 medication classes (n=144) | 73 | 17 | 10 |
| I think one or more of my antihypertensives may currently not be working | | Total sample (n=204) | 63 | 34 | 3 |
|  |  | Using ≥ 2 medication classes (n=141) | 66 | 32 | 2 |
|  | **Concerns about stopping** | | | | |
| I have had a bad experience when stopping one or more of my antihypertensives before | | Total sample (n=187) | 64 | 24 | 12 |
|  |  | Using ≥ 2 medication classes (n=130) | 67 | 23 | 10 |
| I would be reluctant to stop one or more of my antihypertensives | | Total sample (n=206) | 22 | 11 | 67 |
|  |  | Using ≥ 2 medication classes (n=143) | 22 | 12 | 66 |
| With stopping one or more of my antihypertensives I would be worried about missing out on future benefits | | Total sample (n=202) | 25 | 21 | 54 |
|  |  | Using ≥ 2 medication classes (n=140) | 24 | 23 | 53 |
| I get stressed whenever changes are made to one or more of my antihypertensives | | Total sample (n=199) | 71 | 16 | 13 |
|  |  | Using ≥ 2 medication classes (n=138) | 72 | 14 | 14 |
| The recommendation to stop one or more of my antihypertensives makes me feel like my healthcare provider gives up on me | | Total sample (n=202) | 79 | 11 | 10 |
|  |  | Using ≥ 2 medication classes (n=140) | 78 | 11 | 11 |
| n = number of responses | |  |  |  |  |

Appendix 6C. Appropriateness- and concerns-responses about deprescribing **sulfonylurea** (with medication-specific revised Patients’ Attitudes Towards Deprescribing questions)

| **Item** | | **Sample** | **(strongly) disagree (%)** | **Neutral (%)** | **(strongly) agree (%)** |
| --- | --- | --- | --- | --- | --- |
|  | **Appropriateness** | | | | |
| I am positive about stopping my sulfonylurea to see how I feel without it | | Total sample (n=43) | 55 | 5 | 40 |
|  |  | Using ≥ 2 medication classes (n=38) | 58 | 5 | 37 |
| I would agree to reduce the dose of my sulfonylurea | | Total sample (n=42) | 38 | 17 | 45 |
|  |  | Using ≥ 2 medication classes (n=37) | 35 | 19 | 46 |
| I feel that I may no longer need my sulfonylurea | | Total sample (n=42) | 69 | 24 | 7 |
|  |  | Using ≥ 2 medication classes (n=37) | 68 | 24 | 8 |
| I believe my sulfonylurea may be currently giving me side effects | | Total sample (n=43) | 79 | 16 | 5 |
|  |  | Using ≥ 2 medication classes (n=38) | 77 | 18 | 5 |
| I think my sulfonylurea may currently not be working | | Total sample (n=43) | 69 | 26 | 5 |
|  |  | Using ≥ 2 medication classes (n=38) | 66 | 29 | 5 |
|  | **Concerns about stopping** | | | | |
| I have had a bad experience when stopping my sulfonylureabefore *(n=28)* | | Total sample (n=39) | 64 | 33 | 3 |
|  |  | Using ≥ 2 medication classes (n=34) | 62 | 35 | 3 |
| I would be reluctant to stop my sulfonylurea | | Total sample (n=43) | 24 | 9 | 67 |
|  |  | Using ≥ 2 medication classes (n=38) | 21 | 11 | 68 |
| With stopping my sulfonylurea I would be worried about missing out on future benefits | | Total sample (n=43) | 14 | 28 | 58 |
|  |  | Using ≥ 2 medication classes (n=38) | 13 | 26 | 61 |
| I get stressed whenever changes are made to my sulfonylurea | | Total sample (n=43) | 79 | 14 | 7 |
|  |  | Using ≥ 2 medication classes (n=38) | 81 | 11 | 8 |
| The recommendation to stop my sulfonylurea makes me feel like my healthcare provider gives up on me | | Total sample (n=42) | 74 | 12 | 14 |
|  |  | Using ≥ 2 medication classes (n=37) | 72 | 14 | 14 |

n = number of responses

Appendix 6D. Appropriateness- and concerns-responses about deprescribing **insulin** (with medication-specific revised Patients’ Attitudes Towards Deprescribing questions)

| **Item** | | **Sample** | **(strongly) disagree (%)** | **Neutral (%)** | **(strongly) agree (%)** |
| --- | --- | --- | --- | --- | --- |
|  | **Appropriateness** | | | | |
| I am positive about stopping my insulin to see how I feel without it | | Total sample (n=32) | 72 | 9 | 19 |
|  |  | Using ≥ 2 medication classes (n=30) | 70 | 10 | 20 |
| I would agree to reduce the dose of my insulin | | Total sample (n=32) | 63 | 12 | 25 |
|  |  | Using ≥ 2 medication classes (n=30) | 63 | 10 | 27 |
| I feel that I may no longer need my insulin | | Total sample (n=32) | 88 | 12 | 0 |
|  |  | Using ≥ 2 medication classes (n=30) | 90 | 10 | 0 |
| I believe my insulin may be currently giving me side effects | | Total sample (n=32) | 82 | 9 | 9 |
|  |  | Using ≥ 2 medication classes (n=30) | 80 | 10 | 10 |
| I think my insulin may currently not be working | | Total sample (n=32) | 91 | 6 | 3 |
|  |  | Using ≥ 2 medication classes (n=30) | 90 | 7 | 3 |
|  | **Concerns about stopping** | | | | |
| I have had a bad experience when stopping my insulin before *(n=28)* | | Total sample (n=30) | 80 | 20 | 0 |
|  |  | Using ≥ 2 medication classes (n=28) | 79 | 21 | 0 |
| I would be reluctant to stop my insulin | | Total sample (n=32) | 3 | 6 | 91 |
|  |  | Using ≥ 2 medication classes (n=30) | 3 | 7 | 90 |
| With stopping my insulin I would be worried about missing out on future benefits | | Total sample (n=28) | 10 | 11 | 79 |
|  |  | Using ≥ 2 medication classes (n=27) | 11 | 11 | 78 |
| I get stressed whenever changes are made to my insulin | | Total sample (n=32) | 85 | 3 | 12 |
|  |  | Using ≥ 2 medication classes (n=30) | 84 | 3 | 13 |
| The recommendation to stop my insulin makes me feel like my healthcare provider gives up on me | | Total sample (n=31) | 74 | 13 | 13 |
|  |  | Using ≥ 2 medication classes (n=30) | 74 | 13 | 13 |

n = number of responses
